# Neutrophils in COVID-19: recent insights and advances

**DOI:** 10.1186/s12985-023-02116-w

**Published:** 2023-08-02

**Authors:** Jiayu Li, Kegong Zhang, ye Zhang, Ziyang Gu, Changxing Huang

**Affiliations:** Department of Infectious Diseases, Second Affiliated Hospital of Air Force Military Medical University, Xi’an, 710038 China

**Keywords:** COVID-19, Neutrophils, Neutrophil extracellular traps (NETs), Low- density neutrophils (LDNs), Cytokine storm, Thrombosis, Treatment strategies

## Abstract

Coronavirus disease 2019 (COVID-19) is an acute respiratory disease caused by severe acute respiratory syndrome coronavirus 2 (SARS-CoV-2), which can lead to acute respiratory distress syndrome (ARDS), multi-organ failure and death, posing significant threat to human health. Studies have found that pathological mechanisms, such as cytokine storms caused by uncontrolled innate immune system activation, release of damage-associated molecular patterns during tissue injury and a high incidence of thrombotic events, are associated with the function and dysfunction of neutrophils. Specifically, the increased formation of low-density neutrophils (LDNs) and neutrophil extracellular traps (NETs) has been shown to be closely linked with the severity and poor prognosis in patients with COVID-19. Our work focuses on understanding the increased number, abnormal activation, lung tissue infiltration, and elevated neutrophil-to-lymphocyte ratio in the pathogenesis of COVID-19. We also explore the involvement of NETs and LDNs in disease progression and thrombosis formation, along with potential therapeutic strategies targeting neutrophil and NETs formation.

## Introduction

Coronavirus disease 2019 (COVID-19) is a highly contagious respiratory disease resulting from severe acute respiratory syndrome coronavirus 2 (SARS-CoV-2) infection. It was identified as the third lethal coronavirus outbreak in December 2019 after SARS-CoV and the Middle East respiratory syndrome coronavirus (MERS-CoV) [1], and on January 30, 2020, the World Health Organization (WHO) declared it a Global Public Health Emergency [2]. The clinical presentation and prognosis of SARS-CoV-2-infected patients vary greatly, ranging from asymptomatic cases to severe acute respiratory distress syndrome (ARDS) and multiple organ dysfunction syndromes [3]. In addition, considering that the COVID-19 pandemic has had a devastating impact on global health and is primarily driven by dysregulated immune responses, understanding its pathophysiology has been a pressing need since the beginning of the outbreak [4]. Studies have shown that dysregulation of cytokines resulting from uncontrolled activation of the innate immune system, the release of damage-associated molecular patterns during tissue injury, and an increased occurrence of thrombotic events are all associated with the functioning and malfunctioning of neutrophils [5]. Specifically, the increased formation of low-density neutrophils (LDNs) and the generation of neutrophil extracellular traps (NETs) play significant roles in the immunopathology of the disease and are closely correlated with its severity and poor prognosis [5–7].

Neutrophils, the most abundant type of leukocytes in human circulation, account for approximately 50–70% of all leukocytes [8]. Traditionally, neutrophils were believed to primarily function as immediate immune defenders against bacterial and fungal pathogens [9]. However, recent research has revealed that the role of neutrophils is more complex and diverse than previously thought, as they were also found to play a key role in the defense against viral infections, including respiratory syncytial virus (RSV), influenza A virus (IAV), highly pathogenic avian influenza virus, and vesicular stomatitis virus (VSV) [10]. During an infection, pathogen-associated molecular patterns (PAMPs), such as lipopolysaccharide (LPS), lipoteichoic acid, proteins, ribonucleic acid (RNA) and deoxyribonucleic acid (DNA), are released and recognized by the immune system, which then bind to various pathogen recognition receptors (PRRs) to initiate the recruitment of neutrophils to the site of tissue damage [11].

**Fig. 1:**
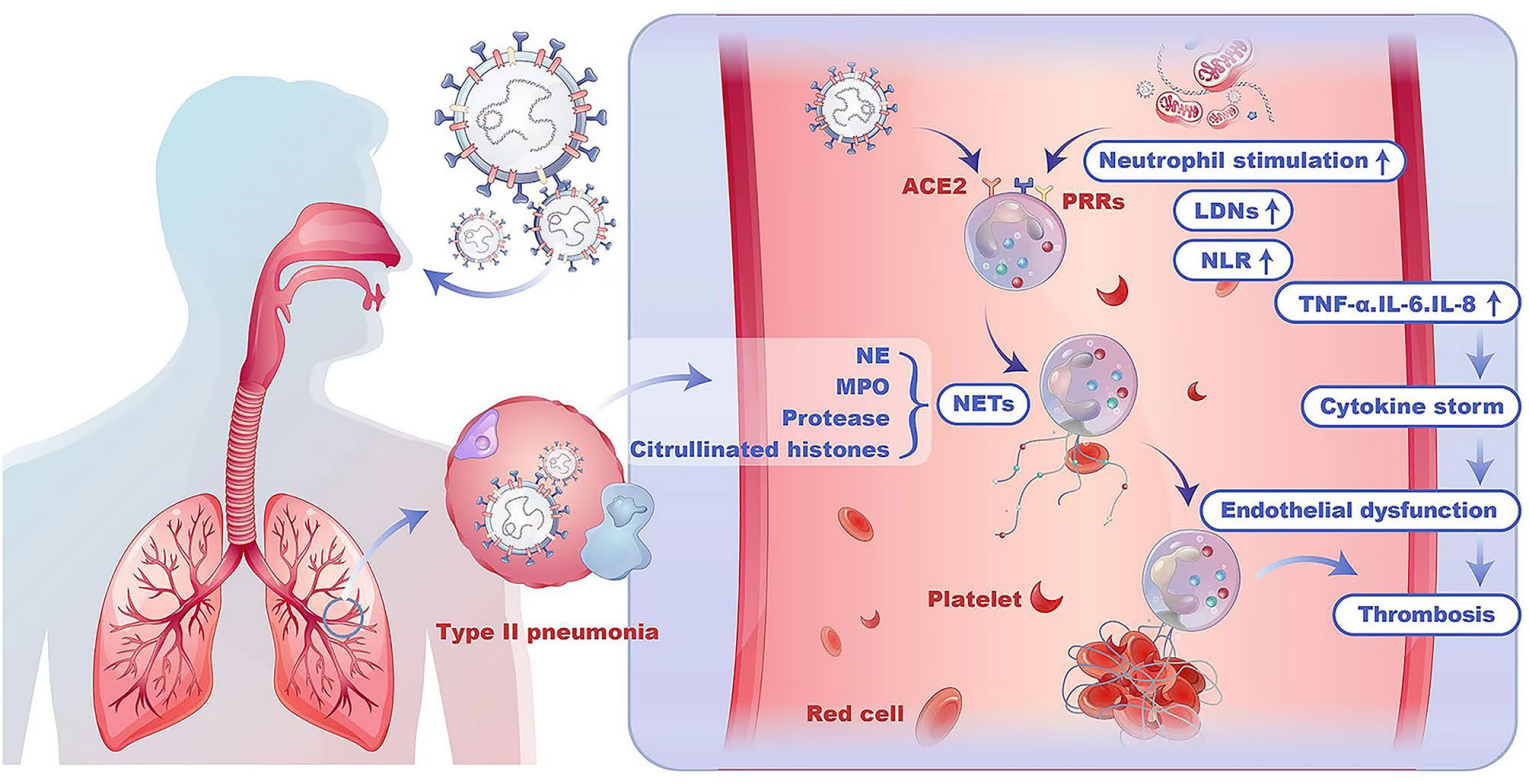
The schematic representation of neutrophils in COVID-19

Activated neutrophils can release a range of pro-inflammatory mediators, such as interleukin-6 (IL-6), interleukin-8 (IL-8), interferon-γ (IFN-γ) and tumor necrosis factor-α (TNF-α) [12, 13]. These mediators play a crucial role in recruiting other immune cells to the site of infection. However, excessive activation of neutrophils can lead to oxidative stress, local and systemic inflammation and subsequent damage to the endothelium of capillaries, contributing to an increased incidence of thrombotic events [3]. (Fig. 1)

**Fig. 2:**
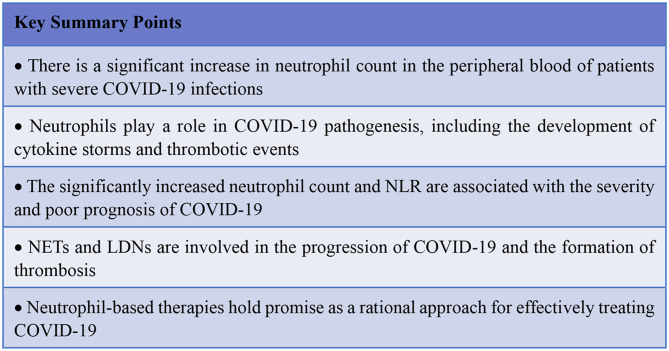
Key summary points

## Neutrophils and COVID-19

Recent studies have observed a significant rise in neutrophil count among patients with COVID-19 [5, 14, 15]. Chen et al. [16] reported that severe or critical COVID-19 patients had notably higher neutrophil counts upon admission than mild/moderate COVID-19 patients. In addition, elevated neutrophil count has been associated with increased disease severity and a poorer prognosis. Similarly, Li et al. observed that a significantly elevated neutrophil count could serve as an indicator to assess disease severity, consistent with the findings of previous reports [17].

The combination of an increased neutrophil count and a decreased lymphocyte count in COVID-19 patients leads to an elevated neutrophil-to-lymphocyte ratio (NLR) [18–20]. Sun et al. [21] compared COVID-19 patients admitted to the intensive care unit (ICU) with non-ICU admitted COVID-19 patients, and found that COVID-19 patients admitted to ICU had the lowest lymphocyte count, the highest neutrophil count and NLR, and the study showed that NLR was an independent predictor of disease severity in patients with COVID-19. Recent studies have identified NLR as an independent predictive marker of disease severity in COVID-19 patients [22–25]. Chen et al. [16] found that the NLR remained significantly higher in non-survivors compared to survivors from admission to the end of hospitalization, further supporting the use of NLR as a reliable prognostic biomarker for early-stage COVID-19.

Additionally, studies have revealed that neutrophil numbers are elevated not only in the peripheral blood but also in the injured tissues. In April 2020, Barnes et al. [26] reported neutrophil infiltration in pulmonary capillaries, neutrophilic mucositis, extravasation into the alveolar lumen and acute capillaritis with fibrin deposition in lung autopsy samples from three COVID-19 patients [27]. Zhou et al. [28] conducted metatranscriptome sequencing and functional analysis of bronchoalveolar lavage fluid (BALF) samples from COVID-19 patients (n = 8), community-acquired pneumonia patients (n = 146) and healthy controls (n = 20) and reported an increased number of neutrophils and upregulation of various neutrophil chemoattractants in COVID-19 patients. Wauters et al. [29] also found elevated neutrophil counts through single-cell deep-immune profiling of BALF samples from patients with mild COVID-19 (n = 5) and critical COVID-19 (n = 26), compared to BALF samples from non-COVID-19 patients. Furthermore, researchers have discovered that in patients with myocardial or liver damage complications, increased neutrophil numbers correlate with markers of tissue damage, such as hypersensitive troponin (hsTnT) [3], alanine aminotransferase (ALT) and aspartate aminotransferase (AST) [11].

## NETs and COVID-19

Neutrophils can release web-like structures called NETs, which capture and immobilize pathogenic microorganisms and produce elevated concentrations of myeloperoxidase (MPO) and defensins to resist exogenous infections [30]. NETs contain a combination of cell-free DNA, citrullinated histones and neutrophil granular proteins [31]. Initially, the formation of NETs was discovered as a response of neutrophils to the presence of bacteria. Interestingly, NETs also possess antiviral defense effects [32]. Their role in combating viral infections has been observed in various diseases, including respiratory syncytial virus (RSV) [33], dengue virus, influenza virus, and even human immunodeficiency virus (HIV) [34, 35]. In addition, multiple studies have reported the involvement of NETs in the immune response against viral pathogens [34, 36].

Studies have reported elevated levels of neutrophil extracellular traps (NETs) in both peripheral blood and lung tissues of COVID-19 patients [14, 37, 38]. In 2020, Barnes et al. reported the involvement of NETs in COVID-19 [27]. Zuo et al. analyzed the serum of 50 infected individuals and demonstrated an increased presence of NETs, indicating their overactivation [39]. Proteomics analysis has revealed an association between granule content, NETs formation capacity and the incidence and severity of respiratory distress in pneumonia patients. Interestingly, elevated NETs components, including citrullinated histones, cell-free DNA, and myeloperoxidase (MPO)-DNA complexes, have also been observed in COVID-19 patients [39]. Guéant et al. [40] conducted a multicenter study involving 155 COVID-19 patients and suggested that neutrophil elastase (NE), deoxyribonuclease (DNase) and NETs are involved in the early and late progression of COVID-19. Among them, the significant elevation of NE and NETs in severe COVID-19 may be closely associated with neutrophil activation through the IL-8/CXCR2 pathway. NE and histone-DNA were found to be related to systemic multi-organ damage, including lung injury, cardiovascular injury and renal insufficiency in COVID-19 patients. NE levels above 593 ng/ml were identified as an independent predictor of multiple-organ damage. Importantly, besides being associated with the increased severity of COVID-19, elevated NETs also contribute to lung injury and microvascular thrombosis [37].

## LDNs and COVID-19

Recent studies have increasingly recognized the heterogeneity of neutrophils, including variations in morphology, phenotype, and function [41]. Neutrophils can be classified into two distinct phenotypes based on their density: LDNs and normal-density neutrophils (NDNs). LDNs are a subset of neutrophilic granulocytes that remain in the peripheral blood mononuclear cells (PBMCs) after density gradient separation. LDNs may arise from the activation and degranulation of mature NDNs and the release of immature neutrophils from the bone marrow. Functionally, LDNs can be further categorized into immunosuppressive LDNs, also known as granulocyte myeloid-derived suppressor cells (G-MDSCs), and pro-inflammatory LDNs, also known as low-density granulocytes (LDGs) [42].

Cabrera et al. reported a significant increase in the frequency of LDNs in the circulating blood of COVID-19 patients compared to age- and gender-matched healthy individuals and an association between LDNs and disease severity, with a particularly pronounced increase in severe COVID-19 patients. In addition, they found that LDNs in COVID-19 patients exhibit phenotypic diversity and possess immunosuppressive properties. Based on their surface marker expression, COVID-19-associated LDNs can be categorized into four distinct subsets: CD33^++^CD16^-^CD11b^-^, CD33^+^CD16^-^CD11b^+^, CD33^low^CD16^+^CD11b^+^ and CD33^-^CD16^+^CD11b^-/low^, which represent different stages of maturation in the development of granulocytes, including promyelocytes, myelocytes, bands and mature granulocytes [43]. Further, the researchers showed that these LDN subsets have immunosuppressive capabilities and may contribute to impaired lymphocyte responses during acute COVID-19 infection [43].

Manunta et al. discovered that an increased number of LDNs in early-stage COVID-19 patients was correlated with disease severity and also observed that the percentage of LDNs exhibited considerable variation, with a mean of 35.4% ± 27.2%. The researchers suggested that LDNs could be degranulated or immature neutrophils released due to bone marrow mobilization [41]. However, transcriptome and proteomics analyses have revealed the presence of a diverse population of LDNs with multiple phenotypes in the peripheral blood of severe COVID-19 patients, indicating the presence of both mature and immature neutrophils in the PBMCs of these individuals [44]. Current studies also support the view that LDNs consist of activated mature neutrophils and numerous immature neutrophils [42, 45], which can be found not only in the circulating blood but also in BALF [44]. Lim et al. observed that the persistence of a substantial number of LDNs during the recovery period was characteristic of the severity of COVID-19 [46]. Importantly, Obermayer et al. found that LDNs were particularly prone to the spontaneous formation of NETs, primarily contributing to the lung injury associated with COVID-19, including vascular obstruction [47].

## Neutrophils, NETs, and thrombosis

An important characteristic of COVID-19 is an increased risk of thrombosis [48]. While the exact mechanisms underlying thrombosis in COVID-19 are still not fully understood, several studies have highlighted the involvement of neutrophils in thromboinflammation associated with the disease [49–51]. ​Based on the role of NETs in other diseases, in April 2020, Barnes et al. [52]and Zuo et al. [53] proposed that the formation of NETs might be related to thrombotic events in COVID-19 patients. Subsequently, Zuo et al. [54] confirmed that elevated levels of neutrophil activation and the formation of NETs were indeed associated with thrombosis in COVID-19. Since then, additional research, including studies by Leppkes et al. [55] and Nicolai et al. [56], has emerged, further supporting the notion that NETs are involved in thrombotic complications associated with COVID-19.

Studies have demonstrated that NETs can promote the formation of thrombosis in a platelet-dependent manner through mechanisms such as platelet adhesion and activation, binding of cells to fibrinogen and von Willebrand Factor (vWF), and direct activation of the coagulation cascade [57]. Apart from its effect on primary hemostasis, NETs also contribute to local thrombin production, thereby increasing the likelihood of clot formation [58]. Furthermore, NETs can initiate thrombosis by activating the extrinsic pathway through tissue factor (TF) production and the contact pathway via the activation of coagulation factor XII (FXII) [57, 59]. Ammollo et al. discovered that the excess extracellular histones associated with NETs have prothrombotic activity by inhibiting thrombin-dependent protein C activation, leading to increased thrombin production [60]. Moreover, NETs have been closely implicated in thrombotic events such as deep vein thrombosis, myocardial infarction, and thrombotic microangiopathy [11].

In hospitalized patients with COVID-19, high levels of plasminogen activator inhibitor-1 (PAI-1) and tissue plasminogen activator (tPA) in the plasma have been found to be closely associated with the count and activation of neutrophils. The interaction between activated neutrophils, platelets, and the coagulation cascade is considered a significant factor in the development of thrombosis. Studies have revealed that platelet-neutrophil aggregates are linked to disease severity and hypercoagulability. In patients with severe COVID-19, platelet-neutrophil aggregates express high TF levels, which is the main trigger of intravascular coagulation and thrombosis [61]. Further, it has also been found that neutrophils can also produce a large amount of TF during COVID-19 infection [57].

Notably, Morrisey et al. proposed that a population of LDNs could correlate with disease severity and hypercoagulability in COVID-19 patients [58], suggesting that LDNs may play a role in the mechanism of thrombosis. Subsequently, Yan et al. conducted peripheral blood transcriptome sequencing and discovered that elevated LDNs could significantly contribute to COVID-19 immunothrombosis ^[62]^.

## Potential therapeutic options for targeting neutrophil and NETs formation

Neutrophils are a potential therapeutic target for COVID-19 patients [21], and recent studies have identified new strategies to reduce neutrophil recruitment and NET formation, which may help alleviate the severity of various lung diseases.

Previously, glucocorticoids were believed to have the ability to reduce neutrophil recruitment and dampen the hyperinflammatory response associated with neutrophils [10, 57]. However, recent studies have indicated that glucocorticoid treatment does not affect neutrophil priming and NET formation [11]. Moreover, the use of glucocorticoids in patients with severe COVID-19 infections may potentially increase blood viscosity. Additionally, glucocorticoids can impact various cells and organs and have significant side effects; thus, it is of great significance to further seek therapeutic agents mainly targeting neutrophils [10, 57].

JAK inhibitors, which can modulate the production of cytokines by neutrophils through Janus kinases, have emerged as potential agents for COVID-19 treatment. Ruxolitinib, a JAK inhibitor, has shown promise in improving clinical outcomes and can be considered for treating COVID-19 patients with respiratory insufficiency and associated ARDS ^[11, 63]^. Cytokine therapy, widely prescribed for various inflammatory and autoimmune diseases, represent another treatment approach. Targeting neutrophil-related cytokines such as IL-1β, IL-1R, IL-6, and IL-17 could be a valuable strategy to enhance the clinical efficacy of COVID-19 [64, 65]. Anakinra, an IL-1β and IL-1R receptor antagonist, is currently being investigated in clinical trials as it has shown the potential to prevent neutrophil accumulation and activation and NET formation. IL-6 receptor antagonists have demonstrated benefits in reducing mortality among high-risk patients in systematic reviews and meta-analyses [66]; thus, targeting IL-6 or its receptors could be a promising therapeutic option for severe COVID-19 cases. Additionally, IL-17 inhibitors have shown potential in attenuating the cytokine storm, and studies indicate that anti-IL-17 monoclonal antibodies can inhibit the excessive inflammatory response triggered by the SARS-CoV-2 virus [67].

​There is an increasing number of studies supporting the inhibitory effects of colchicine on neutrophil recruitment, activation, cytokine production, inflammation and thrombosis [68]. Several clinical studies have been conducted, which have provided further confirmation of the potential benefits of colchicine treatment in patients with COVID-19 by consistently observing that the administration of colchicine could indeed lead to reduced mortality rates and shorter hospital stays in COVID-19 patients [69–72].

Targeting NETs with recombinant human deoxyribonuclease (DNase) could have important therapeutic implications against COVID-19. Exogenous DNase therapy can disrupt NETs’ structure and may compensate for the impaired degradation of NETs due to reduced DNase levels and activity, enhancing NETs clearance [40, 73]. Currently, clinical trials are underway to assess the safety and effectiveness of recombinant human DNase I in treating COVID-19 [10]. In addition, it is important to note that DNase I has a limited impact on the pro-inflammatory components of NETs, such as histones and elastase. Another potential therapeutic approach is the use of disulfiram, which can inhibit NETs formation and downregulate innate immunity and complement/coagulation pathways [74], and has shown the potential to improve the survival of COVID-19 patients [75].

## Conclusion

In summary, although neutrophils are implicated in viral clearance in SARS-CoV-2, excessive neutrophil recruitment and activation can mediate the development of cytokine storms and the amplification of thrombosis in COVID-19. The significantly increased neutrophil count and NLR are linked to increased disease severity and poor prognosis, making them promising biomarkers for monitoring the severity and progression of COVID-19. In addition, NETs and LDNs are involved in the immunopathological processes of COVID-19 and can serve as early indicators of disease progression. Targeting neutrophils and inhibiting the excessive increase of neutrophils and formation of NETs could alleviate inflammatory burdens and reduce mortality in COVID-19 patients. LDNs also hold potential as a therapeutic target, and further research is needed to explore the immune functions of LDN subsets. Overall, this study provides a theoretical basis for better understanding the role of neutrophils in the pathogenesis of COVID-19 and identifying potential new therapeutic targets. Further research on LDNs will contribute to our understanding of neutrophils’ involvement in COVID-19. In addition, therapies targeting neutrophils hold promise as a viable approach for effectively treating COVID-19.

## Data Availability

Not applicable.

## Abbreviations

COVID-19: Coronavirus disease 2019
SARS-CoV-2: Severe acute respiratory syndrome coronavirus 2
MERS-CoV: Middle East respiratory syndrome coronavirus
WHO: World Health Organization
ARDS: Acute respiratory distress syndrome
LDNs: Low density neutrophils
NETs: Neutrophil extracellular traps
RSV: Respiratory syncytial virus
IAV: Influenza A virus
VSV: Vesicular stomatitis virus
PAMPs: Pathogen-associated molecular patterns
LPS: Lipopolysaccharide
RNA: Ribonucleic acid
DNA: Deoxyribonucleic acid
PRRs: Pathogen recognition receptors
IL-6: Interleukin-6
IL-8: Interleukin-8
IFN-γ: Interferon-γ
TNF-α: Tumor necrosis factor-α
BALF: Bronchoalveolar lavage fluid
hsTnT: Hypersensitive troponin
ALT: Alanine aminotransferase
AST: Alanine aminotransferase
MPO: Myeloperoxidase
HIV: Human immunodeficiency virus
NE: Neutrophil elastase
DNase: Deoxyribonuclease
NDNs: Normal density neutrophils
PBMCs: Peripheral blood mononuclear cells
G-MDSCs: Granulocyte myeloid-derived suppressor cells
LDGs: Low-density granulocytes
PAI-1: Plasminogen activator inhibitor-1
tPA: Tissue plasminogen activator
MMP-9: Metalloproteinase-9
TF: Tissue factor
DIC: Diffuse intravascular coagulation
Vwf: Von Willebrand Factor
FXII: Factor XII
JAKs: Janus kinases
